# Water Absorption Properties of Geopolymer Foam after Being Impregnated with Hydrophobic Agents

**DOI:** 10.3390/ma12244162

**Published:** 2019-12-11

**Authors:** Hiep Le Chi, Pavlína Hájková, Su Le Van, Petr Louda, Lukáš Voleský

**Affiliations:** 1 Department of Material Science, Faculty of Mechanical Engineering, Technical University of Liberec, Studenstká 2, 461 17 Liberec, Czech Republiclongsuvp90@gmail.com (S.L.V.); petr.louda@tul.cz (P.L.); lukas.volesky@tul.cz (L.V.); 2 Unipetrol Centre for Research and Education, Revoluční 84, 400 01 Ústí nad Labem, Czech Republic

**Keywords:** geopolymer foam, Lukosil M130, Lukofob ELX, water absorption, water uptake, water absorption coefficient, flexural strength, compressive strength

## Abstract

Geopolymer foam is classified as a lightweight material with high porous in its matrix which has great offer for applications requiring fire-resistant, thermal, and acoustic properties. However, the high sensitivity to humid environments can be a major barrier of geopolymer foam that limits the variety of applications of this material. Based on this drawback, two types of hydrophobic agent (Lukosil M130 and Lukofob ELX) were used as an impregnator to treat the surface of geopolymer foam samples. This paper presented the results of water absorption properties of the untreated and treated geopolymer foam composites. The obtained properties were flexural strength, compressive strength, density, total water absorption, the rate of water absorption, and water absorption coefficient. The results showed that the samples after being impregnated with hydrophobic agents improved significantly their waterproof property especially using Lukosil M130. Moreover, the samples treated with Lukosil M130 had positive impact on their mechanical strength.

## 1. Introduction

The term ‘’geopolymer’’ introduced by Davidovits in the 1970s [1] is the inorganic aluminosilicate polymers which is produced by a combination of rich source materials in silica and alumina such as metakaolin, fly-ash, blast furnace slag, etc., with strongly alkali activators [2,3,4,5,6]. When considering a proper mixing ratio of raw materials, type and concentration of alkaline activator geopolymers can exhibit a wide variety of desired properties and characteristic such as high compressive strength, low shrinkage, high temperature resistance, acid resistance, and fire resistance up to 1200 °C, etc., [7,8,9,10,11,12,13]. In recent years, this type of material has become an attractive topic in research for the reason that geopolymer concrete offers great potential for alternative to Portland cement-based concrete because of the issue of CO_2_ emission in production of the Portland cement causing the environment pollution [14,15].

Among many products that are established on geopolymer binders, geopolymer foam belongs to the class of lightweight materials with high porous in its structure which has great use in some areas of the construction requiring fire-resistant, thermal, and acoustic insulation properties [16,17,18,19,20,21,22,23]. It is well-known that geopolymer foam is formed from solid geopolymers with addition of foam agents such as metals (silica, alumina, zinc powders), H_2_O_2_, etc., thus pure geopolymer foam is very sensitive to brittle fracture because of the large number of void spaces in structure. The addition of various types of the fillers including particles and fibers will help to improve significantly the mechanical properties of geopolymer foams [19]. However, the existence of fillers especially fiber reinforcements in matrix has a strong influence on the long-term durability of the composite when considering the weathering aspect. The structural performance of geopolymer foam makes it very sensitive to humidity because of easy water ingress through the highly porous structure. The water carrying aggressive agents in the environment, such as H_2_O, O_2_, CO_2_, Cl^−^, SO_4_, cause reinforcement corrosion leading to the deterioration of the structures, such that its service-life is reduced.

Microstructure of geopolymers defines the possibility for water ingress in the material, and therefore microstructure is a key criterion in geopolymer long-term durability. The high sensitivity to humid environments can be considered as a major barrier of geopolymer foams that limits the variety of applications of this material. Therefore, the surface treatment of these composites should be adapted to increase their service life. There are three basic approaches used in applying surface protection to concrete [24]: (i) Hydrophobic impregnation is to produce a water-repellent surface, and the pores and capillaries are internally coated, but they are not filled; (ii) impregnation is to reduce the surface porosity by filling partially or totally the concrete pores; (iii) coating is to produce a continuous protective layer on the concrete surface. Some hydrophobic agents can ingress deep into the inner structure and fill a large number of matrix pores resulting in reduction of surface porosity and improvement of mechanical strength of the treated concrete. The others produce a pore lining effect or form a protective layer at the concrete surface, which acts as a barrier to prevent and/or delay the water penetration.

In the previously reported findings, many researches have been performed on the techniques, various raw materials, foam agents, etc., to achieve improved physical-mechanical properties of geopolymer foam such as fire resistant, thermal, and acoustic properties. In this work, the surface impregnation treatment will be carried out in order to evaluate the water absorption properties of the geopolymer composites. Two types of hydrophobic agents based on siloxane was used, and geopolymer foam is a composite material which was produced by a combination of geopolymer paste and the fillers such as quartz sand, silica fume, chopped basalt fiber, and foam agent.

## 2. Materials and Methods

### 2.1. Raw Materials

Geopolymer Baucis L_k_, supplied by Ceske Lupkove Zavody, a.s Czech Republic, was used as the aluminosilicate source for producing geopolymer paste (in weight percent: SiO_2_—47.4; Al_2_O_3_—29.7; CaO—14.5; MgO—2.6; TiO_2_—1.8; Fe_2_O_3_—0.5; K_2_O—0.3; Na_2_O—1) along with potassium silicate activator of modul 1.71 (in weight percent: SiO_2_—19.56; K_2_O—17.87; H_2_O—62.57). In order to prepare geopolymer paste, the mixing ratio of two components (solid, liquid) was taken out according to the requirement of the manufacturer.

Quartz sand with brand name ST 03/08, supplied by Sklopisek Strelec a. s. Czech, was used as the fine aggregates for the geopolymer mortar matrix (grain size: 0.315–0.80 mm). Powder additive (microsilica) based on amorphous SiO_2_ for concrete and mortar was purchased from Kema Mikrosilika–Sanační centrum s.r.o., Sviadnov Czech Republic. The chemical composition of microsilica as follows (wt. %): SiO_2_—90, CaO—0.8, MgO—max. 1.5, Al_2_O_3_—max. 1, Na_2_O—0.5. This additive was added into geopolymer mortar to enhance both the workability of the fresh mortar and mechanical strength of the hardened mortar. The chopped basalt fiber (BF) was provided by Kamenny Vek, and the tows were about 6.4 mm long with the individual fiber diameters of 13 µm, the density of 2.67 g/cm^3^, tensile strength in the range of 2700–3200 MPa, and tensile modulus of 85–95 GPa. An aluminum powder, supplied by Pkchemie Inc., Czech Republic, was used to make geopolymer foam.

Two different types of the hydrophobic agents with the commercial names Lukosil M130 and Lukofob ELX, provided by Lucebni zavody Kolin a.s., Czech Republic, were used as impregnator for the geopolymer foam samples. Lukosil M130 product is a transparent solution of methyl-silicon resin in xylene, in which the presence of methyl group in the polysiloxane chain makes it hydrophobic with density of 1000–1020 kg/m^3^, viscosity of 30–40 mPa.s/20 °C, heat resistance of max. 230 °C. Its thermal curing enhances hardness and marked improvement in mechanically and mainly chemically resistant film. After drying, it forms a thin, non-stick, heat and weather-resistant film. Lukofob ELX product is a milk-white aqueous emulsion of methyl-silicone resin designed for the final surface waterproof impregnation of porous or less porous silicate materials, which has density of 1000–1010 kg/m^3^, viscosity of 60–80 mPa.s/20 °C [25,26].

### 2.2. Preparation of Geopolymer Foam Composite

The raw materials and mixing ratio for producing geopolymer foam composite are shown in Table 1. Geopolymer mortar was prepared as the following steps. First, geopolymer cement and activator with a given ratio were mechanically stirred for about 3 min to gain homogenous geopolymer paste. Second, silica fume was added to the slurry and mixture was stirred for about 2 min more. Next, rough quartz sand along with chopped basalt fiber was added to prepared mixture followed by mixing for a few minutes until ensuring a homogenous mortar. Finally, Al powder was added into prepared mixture and stirred for about 1 min to create pores inside the geopolymer composite. It should be said that the mixing ratio used in Table 1 was optimized in our lab. The freshly prepared mortar was poured immediately into the molds with a dimension of 40 × 40 × 160 mm^3^. After casting, all the specimens were wrapped using a polypropylene film, and cured at room temperature, ~22 °C, with 45% relative humidity for 24 h. Afterward, the specimens were demolded, and wrapped again using a polypropylene film, and kept at room temperature until 7 days. Finally, the specimens were unwrapped and put into the drying oven at the temperature of 70 °C until an unchanged weight is reached so that they can be used to impregnate with silicone solutions.

### 2.3. Impregnation Process of the Samples with Silicone Solutions

**Figure 1:**
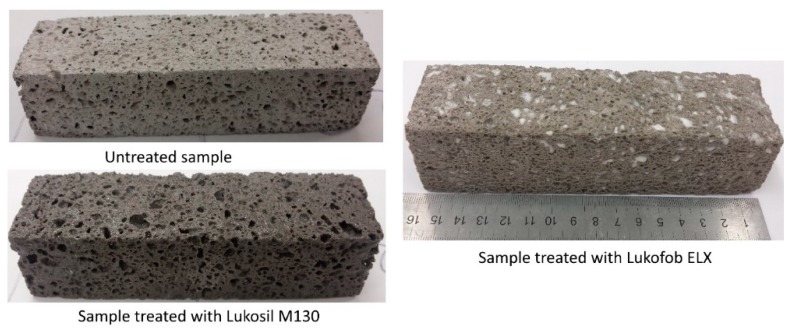
Photographs of the samples without and with impregnation of the silicone solutions.

Before performing this step, all the specimens were carefully weighed. Because of the large porous size of the geopolymer foam, the impregnation approach (wet out) was selected to easily apply the silicone solutions to all surfaces of the geopolymer foam specimens. The specimens prepared in the previous step were placed in a clean bath followed by pouring the silicone solution into the bath until the specimens are immersed in solution to a depth of 20 mm. It should be noted that during immersion the bottom surface of the samples was changed to ensure that the solution is uniformly absorbed into the matrix at all surfaces. They were left for about 30 min to ensure the visible pores in matrix are completely drenched with the solution. The specimens are then removed and drained for about two hours before being placed in the drying oven at the temperature of 70 °C for 48 h. All specimens are removed out of the oven after 48 h and weighed again. The final sample of the impregnation process can be clearly seen in Figure 1. It was found that it consumed about 45.92 g of Lukosil M130 to impregnate each specimen, whereas for Lukofob ELX is about 22.38 g, and this consumed value is calculated only after the impregnated specimens were dried in the oven.

### 2.4. Geopolymer Foam Composite Estimation

The water absorption test of the specimens was performed to know the total water absorption capacity of untreated and treated geopolymer foam. It was determined in accordance with ČSN EN 13755 standard. The samples after being dried to constant mass in the early step were placed in a container of boiled water (to remove dissolve gases) at a room temperature of ~22 °C with a level reaching half the height. Water is gradually added after 1 h to ¾ of the height, after 2 h to complete immersion to a depth of 25 ± 5 mm. The samples are removed from the water after 48 h, wiped with a damp cloth, and weigh quickly. The test was measured for five samples and an average value of measurements was taken. The total water absorption is calculated as per the Equation (1):

(1) $$A=\frac{m_{w}-m_{d}}{m_{d}}\times 100\;\left(\%\right)$$

where *A* is total water absorption (%), *m_d_* is mass of oven-dried sample in air in gram, *m_w_* is mass of the sample saturated with water in gram.

The determination of the water absorption coefficient by capillary action is performed according to ČSN EN 1925. The samples with a dimension of 40 × 40 × 40 mm^3^ that were cut from 40 × 40 × 160 mm^3^ samples were weighed and the area of the submerged base is calculated and expressed in square meters. The sample is placed on thin pads in a closed container so that only a part of the base rests on them. The base was immersed in water to a depth of 3 mm and the water level during test was kept constant. The amount of absorbed water is found out through the weight of the sample in a period of time throughout the contact with water. The mass of water soaked in grams divided by the area of the immersed base of the sample in square meters as a function of the square root of time expressed in seconds is expressed by the graph. The water absorption coefficient was defined as equivalent to the slope of the linear regression line of the first part of the graph.

The flexural strength and compressive strength tests were applied to evaluate the mechanical properties of the geopolymer foam specimens, in which the test method is conducted according to EN 196-1 [27]. The testing machine with load cell capacity of 100 kN (FP Lab Test II, from LABORTECH s.r.o. Opava, Czech Republic), located at the Technical University of Liberec Laboratory, with the applied load under displacement control at a loading rate of 4 mm/min, was used. The mechanical strength of the specimens was tested as the following procedure: (i) The samples with label D-sample mean that they were dried at 70 °C in oven to constant mass in order to perform water absorption tests, as mentioned early; (ii) the samples with label D-W-sample mean that the number of the samples were selected in step 1 and then soaked in water for 48 h; (iii) the samples with label D-W-D-sample mean that the number of the samples were selected in step 2, then they were again dried in oven at 70 °C to constant mass. In other words, the samples that were impregnated by Lukosil M130 are named the LS-sample while those were impregnated by Lukofob ELX are named the LF-sample. Five samples for each recipe was tested and an average value of measurements was taken.

## 3. Results and Discussion

**Figure 2:**
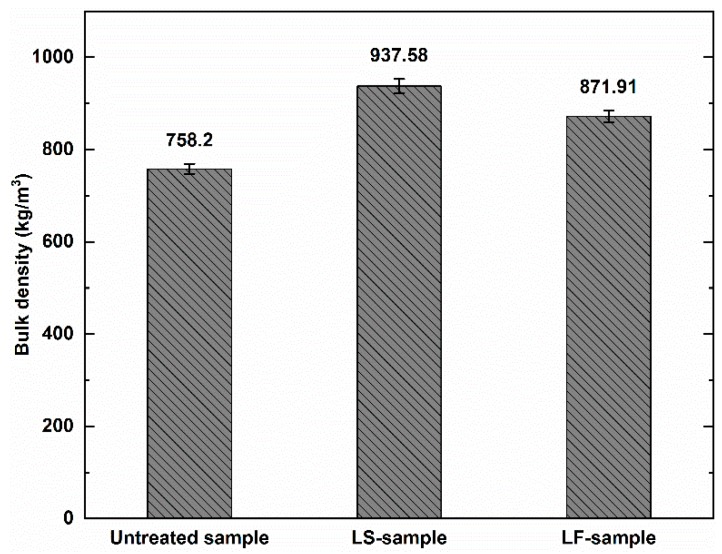
Bulk density value of the geopolymer foam composites.

Figure 2 shows the influence of the impregnators on bulk density of the geopolymer foam composite samples. The bulk density value of the reference sample was 758.2 kg/m^3^ which increased by 23.66% and 14.99% when the specimens were impregnated by Lukosil M130 and Lukofob ELX solutions, respectively. The higher value of bulk density in LS-sample can be explained by the fact that Lukosil M130 has significantly lower viscosity compared to Lukofob ELX, which can be attributed to better penetration into geopolymer matrix leading to greater bulk density. Moreover, Lukosil M130 uses xylene liquid as a solvent, which attributed to the superior dissolving of methyl-silicone component in solution resulting in better ingress into the matrix pores. On the other way Lukofob ELX uses water as a solvent which results in largecluster size of methyl-silicone resin because of its insolubility in water leading to poor ingress into the matrix pores.

**Figure 3:**
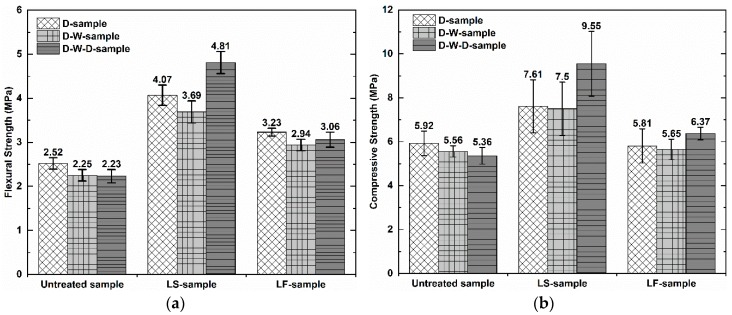
Mechanical properties of the geopolymer foam composites: (**a**) flexural strength; (**b**) compressive strength.

Figure 3 shows the flexural strength and compressive strength of the geopolymer foam composite samples. It can be observed that when all samples were saturated in water after 48 h of immersion, they did not show a significant reduction in mechanical strength compared to dry samples. The average mechanical strength of the untreated samples is 2.52 MPa in flexural and 5.92 MPa in compressive strength. The average mechanical strength of the LS-samples and LF-samples increases by 61.51% (4.07 MPa), 28.55% (3.23 MPa) in flexural and 28.17% (7.61 MPa), −1.85% (5.81 MPa) in compressive strength, respectively, compared to untreated samples. It showed that the LS-samples show a marked improvement in their mechanical strength, while the LF-samples have the enhanced flexural strength value, compared to the untreated those. The improved mechanical strength of the LS-samples can be explained by the fact that the greater precipitation of the methyl silicone components in the matrix acts as a reinforcing agent, contributing to enhanced mechanical strength. Moreover, the LS-samples showed an increase in mechanical strength again when they were oven-re-dried after immersion in water. This finding can be said that it seems that the initial oven-dry mode at 70 °C for 48 h of the LS-samples after impregnation is not effective enough for Lukosil M130 to fully utilize its effects, as a result of the maximum unsatisfactory mechanical strength of those samples. The effect of Lukosil M130 on the marked improvement in mechanical properties of the substrates is clearly mentioned by the manufacturer if they are reasonably cured at high temperatures [24].

**Figure 4:**
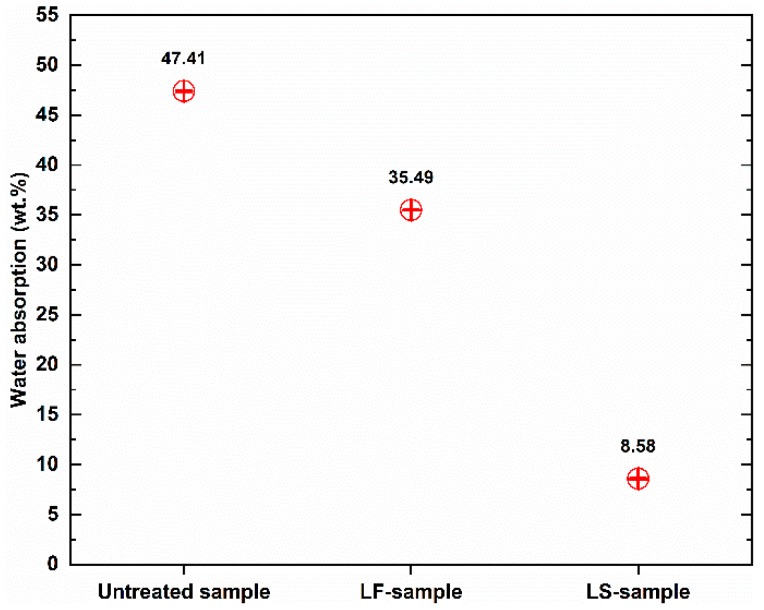
Total water absorption of the geopolymer foam composites.

Figure 4 shows the total water absorption capacity of the geopolymer foam composite samples after 48 h of immersion in water. It is notable that the water-resistant performance of treated geopolymer foam was improved significantly, especially those treated with Lukosil M130. The total water absorption of untreated samples is 47.41%, which significantly decreased by 25.14% and 81.90% when the samples were treated with Lukofob ELX and Lukosil M130, respectively.

**Figure 5:**
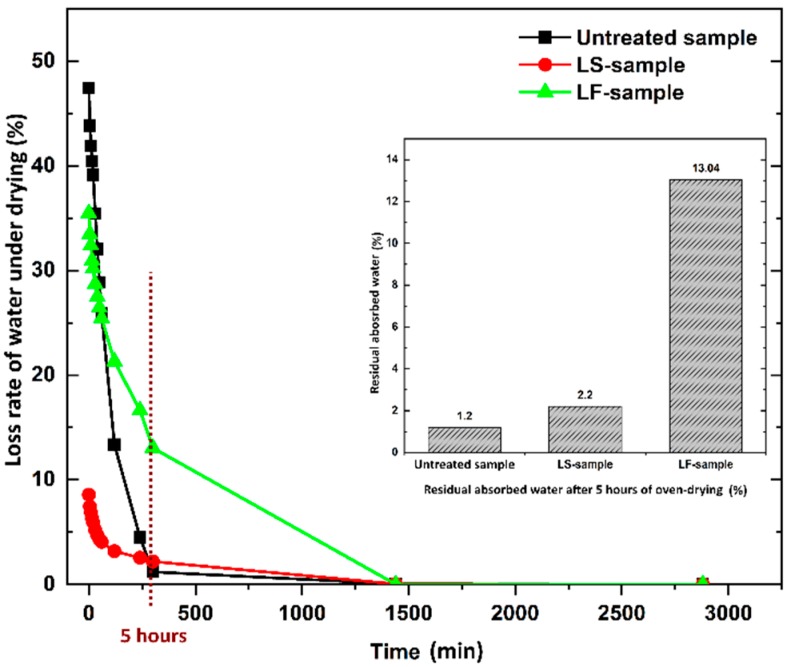
The rate of water loss during the oven-dried process of the geopolymer foam composite.

The loss rate of absorbed water of the samples under oven-drying is shown in Figure 5. It is found that the amount of absorbed water is released from the samples very quickly during the first 2 h of the oven-drying process, and the geopolymer foam samples almost achieve constant weight after 5 h of oven-dried, except for the LF-samples. The residual percentage of water after this duration was 1.2%, 2.2%, and 13.04%, respectively for the untreated samples, LS-samples, and LF-samples. This is attributed to the penetration of hydrophobic agent of Lukofob ELX caused by its material characteristics. Lukofob ELX is an aqueous emulsion with high viscosity, which limits good penetration into pore spaces and deep transportation into matrix. Moreover, the pores at the surface layer of the samples are sealed partially (see in Figure 1). Thus, a barrier in the surface layer is formed which hinders the water penetration and migration. As a result, it takes longer time for water to escape from the material. In the contrast, because of the complete dissolving of methyl silicone in xylene solvent, the hydrophobic agent of Lukosil M130 is transported deeper into the matrix and fills in a greater number of matrix pores as well, as a result of better elimination of water absorption capacity. This result is consistent with the results of the bulk density and mechanical strength performance of the LS-samples and LF-samples.

**Figure 6:**
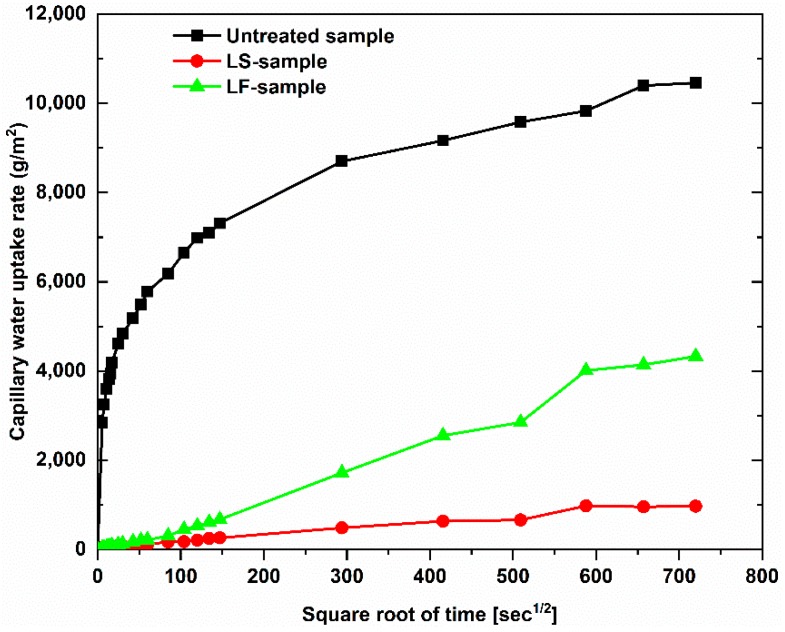
Capillary water uptake for geopolymer foam composite with different service duration.

**Figure 7:**
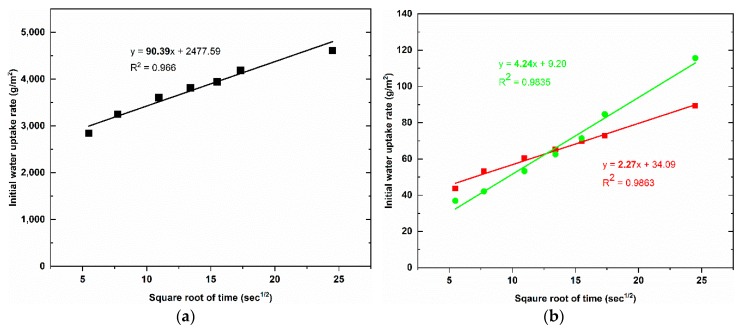
Water uptake rate of the geopolymer foam samples at the first period of 0.5 min to 10 min: (**a**) the untreated samples; (**b**) the LS-sample (red line) and the LF-sample (green line).

**Figure 8:**
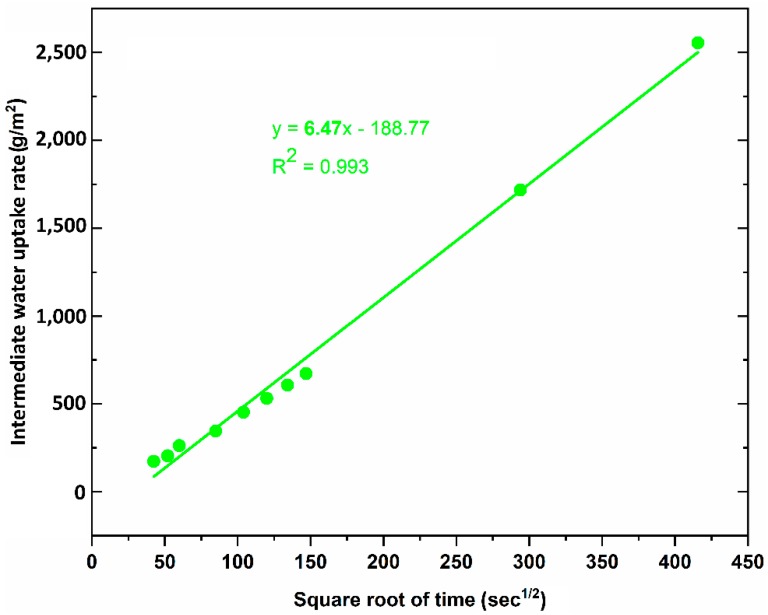
Water uptake rate at the intermediate period of 30 min to 2 h of the LF-sample.

Figure 6 shows the results of capillary water uptake curves with different service duration, whereas the water uptake rate at the initial period is indicated in Figure 7, and the results of the water uptake coefficient of geopolymer foam composite are listed in Table 2. It can be observed that there is a large reduction in capillary water uptake of samples treated with two types of hydrophobic agents, despite high open porosity in geopolymer foam. The untreated samples quickly absorbed a large amount of water during test, and the high rate of capillary water uptake happened in the initial period and this degree was gradually decreased over time. For the LF-samples the low rate of capillary water uptake occurred in the initial period but then increased in the intermediate period and finally a reduction of the water uptake rate exhibited for further periods. This phenomenon is consistent with the above explanation regarding the slow loss rate of water under oven-dried process. The initial capillary uptake is controlled by the surface layer of the sample which acts as a barrier that hinders water penetration. However, once the water gets over this obstacle, an observed rapid increase of capillary suction because of water requirement of the inner pores can be seen in the intermediate period of 30 min to 2 h. Eventually a subsequent decrease of the capillary suction indicated for the longer periods because of higher water content in the interior of the sample and the slow progressive participation of the less accessible pores [28]. The rate of capillary water uptake of the LS-samples is lower in the initial period compared to the LF-samples and tends to reach saturation after 6 days of test. The water absorption coefficient is defined as equivalent to the slope of linear regression line of the initial period of the graph which is selected from 0.5 min to 10 min (Figure 7). However, because of the higher capillary suction in the intermediate period, the authors think that the water absorption coefficient of the LF-sample should be mentioned over the period of time that ranges from 30 min to 2 h (Figure 8). The water absorption coefficient of the untreated samples is 90.39, which decreased significantly by 97.49% when the samples are treated by Lukosil M130 (Table 2). On the other hand, it decreased marked by 95.31% and 92.84% when the LF-sample considered in the initial and intermediate period, respectively (Table 2). It is observed that capillary water uptake test revealed better waterproofing ability of the LF-samples compared to test method of total water absorption, in which the samples were immersed in water up to full saturation.

## 4. Conclusions

In the present research, the geopolymer foams were treated with two types of hydrophobic agent at the age of 7 days after casting and their physical-mechanical properties such as flexural strength, compressive strength, bulk density, water absorption capacity, and water absorption coefficient were analyzed. Based on the experimental results achieved, the following conclusions are outlined: The higher bulk density of the LS-sample revealed that the hydrophobic agent of Lukosil M130 filled in the greater number of pores in the matrix leading to better physical-mechanical properties of the geopolymer foam composite compared to that of Lukofob ELX. Moreover, using Lukosil M130 for impregnating samples also helps significantly in the improvement of mechanical strength of geopolymer foams if they are reasonably cured at high temperatures.

The LF-samples indicate that their total water absorption capacity is much higher than that of LS-sample. The LS-samples with water absorption capacity of 8.58% decreased significantly by 81.90%, while the LF-samples with this value of 35.49% decreased by 25.14%, compared to untreated samples with value of 47.41%. However, under capillary water uptake test, the water absorption coefficient of the LF-samples was quite good compared to that of the LS-samples. The untreated sample obtained water absorption coefficient with 90.39 while this value was 6.47 and 2.27 for LF-sample, LS sample, respectively.

Lukosil M130 has proved that it is an excellent hydrophobic agent for geopolymer foam as it is applied by the impregnation method. The authors recommend that further work is required to find out the optimal oven-dried regime (temperature and time) of the LS-sample which influences significantly on their mechanical strength. Moreover, the preliminary results obtained in this work were tested at the early-age impregnated samples. Therefore, the long-term performance of waterproof property of geopolymer foam treated with Lukosil M130 and Lukofob ELX should be considered. On the other hand, Lukofob ELX is considered as a hydrophobic agent with high environmental friendliness.

## Figures and Tables

**Table 1:** Mixture of geopolymer foam composite.

By wt.% of Geopolymer Cement			By Weight Ratio (-)		
BF content	Silica fume	Al powder	Geopolymer cement	Activator	Silica sand
15	5	1.5	1	0.9	1

**Table 2:** Water absorption coefficient of geopolymer foam composites.

No. Sample	Water Absorption Coefficient (g/m^2^/s^0.5^)	Correlation Coefficient (R^2^)
Untreated sample	90.39	0.97
LF-sample	(Initial) 4.24	0.98
	(Intermediate) 6.47	0.99
LS-sample	2.27	0.99
